# Association between initial intravenous fluid volume and the composite outcome of hemodialysis dependence at discharge or in-hospital mortality in inpatients with rhabdomyolysis

**DOI:** 10.1186/s40560-025-00788-w

**Published:** 2025-04-27

**Authors:** Wataru Yajima, Shotaro Aso, Hiroki Matsui, Kiyohide Fushimi, Hideo Yasunaga

**Affiliations:** 1 Department of Clinical Epidemiology and Health Economics, School of Public Health, University of Tokyo, 7-3-1 Hongo, Bunkyo-Ku, Tokyo, 1130033 Japan; 2 Department of Emergency Medicine, Jikei University School of Medicine Kashiwa Hospital, Chiba, Japan; 3 Department of Health Policy and Informatics, Tokyo Medical and Dental University Graduate School of Medicine, Tokyo, Japan

**Keywords:** Acute kidney injury, Fluid resuscitation, Hemodialysis, In-hospital death, Intravenous fluid, Rhabdomyolysis

## Abstract

**Background:**

Although experts recommend intravenous fluid (IVF) for patients with rhabdomyolysis to prevent renal injury, the optimal IVF volume remains unknown because excessive IVF may lead to organ edema, resulting in organ injury. This study aimed to investigate the association between IVF volume and the composite outcome of hemodialysis dependence or in-hospital death in patients with rhabdomyolysis.

**Methods:**

We retrospectively identified patients with rhabdomyolysis admitted to intensive care units and tertiary-care hospitals from July 2010 to March 2022 using the Japanese Diagnosis Procedure Combination database. We divided the patients into those who received at least 3500 mL/day of IVF within 3 days of admission and those who did not. This threshold was defined based on the findings of previous studies. We compared the composite outcome, including hemodialysis dependence at discharge and in-hospital death, between the groups using propensity score overlap weighting.

**Results:**

We identified 5392 eligible patients. Of those, 1677 (31.1%) received ≥ 3500 mL/day of IVF, and 3715 (68.9%) received < 3500 mL/day of IVF; the total volumes of IVF within 3 days of admission were 11,039 mL and 4054 mL, respectively. Propensity-score overlap weighting created balanced cohorts, which did not show significant difference in primary composite outcome between the groups (3.4% vs. 3.9%; risk difference [RD] − 0.4, 95% confidence interval [CI] − 1.8 to 0.9, P = 0.53). The proportion of hemodialysis dependence was lower in the IVF ≥ 3500 mL/day group than those in the < 3500 mL/day group (0.4% vs. 1.3%; RD − 0.9, 95% CI − 1.6 to − 0.2, P = 0.02).

**Conclusions:**

This retrospective database study found that IVF ≥ 3500 mL/day for patients with rhabdomyolysis was not associated with a reduction in the composite outcome but was associated with a reduction in hemodialysis dependence at discharge. The optimal IVF volume still remains unknown, and further studies are warranted.

**Supplementary Information:**

The online version contains supplementary material available at 10.1186/s40560-025-00788-w.

## Background

Rhabdomyolysis is characterized by the destruction of skeletal muscles and the entry of intracellular enzymes into the blood. Especially, myoglobin causes renal tubular necrosis through various pathways, leading to acute kidney injury (AKI) in about 20–50% of patients with rhabdomyolysis [1]. Mortality in patients with AKI in the intensive care unit (ICU) was higher than that in those without AKI (60% vs. 20%) [2, 3]. Rhabdomyolysis is a potentially fatal syndrome, and it is crucial to prevent patients from developing AKI. One treatment strategy for rhabdomyolysis is adequate fluid replacement to increase the clearance of nephrotoxic substances [4]. To date, no randomized controlled trials or systematic reviews have assessed the association between IVF volume and the incidence of AKI after rhabdomyolysis. Based on previous observational studies [5–7], more than 6000 mL of intravenous fluid (IVF) per day was recommended [1, 8]. In contrast, one study found that > 3600 mL of IVF per day for rhabdomyolysis resulted in increased renal injury [9].

However, clinicians may believe that the recommended volume of IVF (> 6000 mL) is excessive. A previous study reported that 64% of the patients with rhabdomyolysis received less than 2000 mL/day [10]. The optimal IVF volume for rhabdomyolysis remains to be elucidated.

We set a threshold for the amount of IVF at 3500 mL and hypothesized that IVF ≥ 3500 mL per day was excessive and potentially harmful. The reason for setting the threshold at 3500 mL/day was that many patients actually receive IVF < 2000 mL/day in a real-world clinical setting, and IVF > 3600 mL/day might be harmful, according to the previous study [10].

The present study investigated the association between IVF volume of ≥ 3500 mL/day and the composite outcome of hemodialysis dependence at discharge or in-hospital death in patients with rhabdomyolysis, using a national inpatient database in Japan.

## Methods

### Data source

This retrospective cohort study utilized the data from the Japanese Diagnosis Procedure Combination (DPC) inpatient database [11]. This database includes administrative claims data and discharge abstracts from more than 1200 acute-care hospitals and covers approximately 90% of all tertiary-care hospitals in Japan. We could obtain patient data, such as age, sex, height, weight, consciousness, ICU admission, and in-hospital mortality. Diagnoses and comorbidities at admission and complications during hospital stay were recorded using the International Classification of Diseases, Tenth Revision (ICD-10) codes and text data in Japanese. It also provided information related to procedures, such as the volume of IVF, vasopressors, prescriptions, dialysis, oxygenation, and mechanical ventilation.

The database has been well-validated, and the specificity of diagnosis was reported to exceed 96%, with the sensitivity of 50–80%. The sensitivity and specificity of the procedure were over 90% [12].

### Study cohort

We identified patients aged ≥ 18 years whose main diagnosis or primary diagnosis at admission was rhabdomyolysis (ICD-10 code: M62.8), drug-induced rhabdomyolysis (Y57.9), and crush syndrome (T79.5), and admitted to ICU or tertiary-care hospital from July 2010 to March 2022. We excluded patients diagnosed with other diseases with M62.8, those discharged within 3 days after admission, and those who depended on dialysis before admission. Dependence on hemodialysis before admission was defined as a comorbidity of chronic kidney disease (CKD) before admission, receipt of dialysis within 2 days after admission.

We divided the eligible patients into two groups: those who received ≥ 3500 mL IVF in a single day within 3 days after admission (the IVF ≥ 3500 mL group) and those who received < 3500 mL IVF in a single day (the IVF < 3500 mL group).

### Outcomes and covariates

The primary outcome was the composite outcome of hemodialysis dependence at discharge or in-hospital death, considering competing risks. Based on previous studies, we defined hemodialysis dependence at discharge as the receipt of hemodialysis within 3 days before discharge [13]. The secondary outcomes were hemodialysis dependence at discharge, in-hospital death and 28-day hospital-free days. The duration of 28-day ventilator-free days or 28-day oxygenation-free days was assessed in those who received mechanical ventilation or oxygenation.

Covariates were as follows: (1) patient characteristics, including age, sex, body mass index, Charlson Comorbidity Index, Japan Coma Scale, comorbidity of chronic renal failure, (2) causes of rhabdomyolysis (trauma, heat stroke, hypothermia, burn, drug intoxication, alcohol intoxication, epilepsy, hypothyroidism, diabetic ketoacidosis, severe asthma exacerbation, neuroleptic malignant syndrome, and infection), (3) procedures performed within 3 days after admission, including crystalloids (mL), maintenance fluid (mL), red blood cell transfusion (mL), plasma transfusion (mL), platelet transfusion (mL), and albumin (mL); use of vasopressors (noradrenaline, dopamine and vasopressin), sodium bicarbonate, diuretics, and lipid-lowering drugs (statins and fibrates); continuous renal replacement therapy; and high flow nasal cannula, mechanical ventilation and oxygen administration via cannula or mask. The Japanese Coma Scale is a widely used measure of impaired consciousness in Japan and correlated with the Glasgow Coma Scale [14].

### Statistical analysis

Continuous variables were described as mean and standard deviation (SD), in addition to median and interquartile range (IQR). Categorical variables were described as numbers and percentages.

To adjust for confounders, we conducted a propensity-score overlap weighting [15–17]. We calculated the propensity score for receiving ≥ 3500 mL of IVF within 3 days from admission using a multivariable logistic regression analysis fitted with a generalized estimating equation to adjust for within-hospital clustering as well as the above variables. The patients receiving ≥ 3500 mL of IVF per day were weighted by 1-propensity score, while those receiving < 3500 mL of IVF per day were weighted by propensity score. Differences between the two groups were presented as standardized differences. The absolute standardized difference < 0.1 was considered well-balanced [18, 19].

We conducted subgroup analyses for the composite outcome, hemodialysis dependence, and in-hospital death stratified by those aged ≥ 65 years or < 65 years, those with and without CKD before admission, those on vasopressors or mechanical ventilation, and those with different causes of rhabdomyolysis (trauma or others).

Three sensitivity analyses were performed. First, the eligible patients were divided into three groups: IVF < 3500 mL, 3500–5999 mL and ≥ 6000 mL. Subsequently, propensity score matching weight analysis was conducted, which allowed us to compare outcomes among the three groups [20]. We calculated three propensity scores for each patient that predicted probabilities of receiving every defined exposure—namely IVF < 3500 mL, 3500–5999 mL, and ≥ 6000 mL—using a multinomial logistic regression. Matching weight was defined as the minimum of the three propensity scores divided by each propensity score that predicted the probabilities of receiving the actual amount of IVF. The IVF < 3500 mL group was set as the reference, and multinomial regression was conducted to compare the groups. Second, the exposure was changed from the amount of IVF (mL) to the amount of IVF per body weight (mL/kg). Considering the standard body weight as 60 kg, the patients were divided into two groups: IVF < 60 mL/kg (IVF < 3600 mL/day equivalent) and IVF ≥ 60 mL/kg (IVF ≥ 3600 mL/day equivalent). Third, considering that normal saline can cause AKI induced by hyperchloremia [21], we conducted a propensity-score overlap weighting analysis, using whether the patients were receiving more than 1500 mL of normal saline per day within 3 days of admission when calculating propensity score.

The two-sided significance level was defined as P < 0.05. All the analyses were performed using Stata version18 (StataCorp, College Station, TX, USA).

## Results

**Fig. 1:**
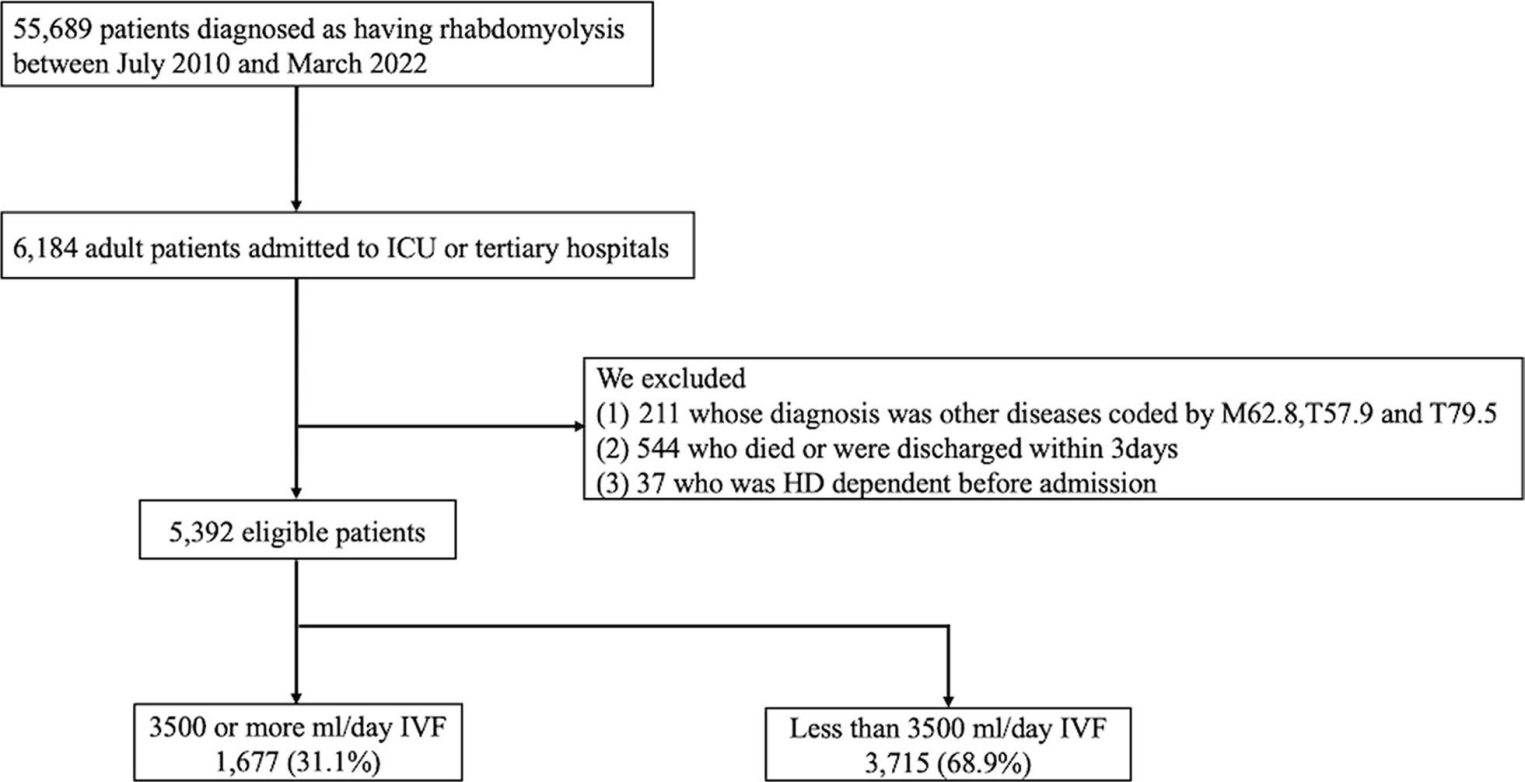
Flowchart of patient selection. *ICU* intensive care unit, *IVF* intravenous fluid, *HD* hemodialysis

Of the 55,689 patients diagnosed with rhabdomyolysis upon admission during the study period, 6184 were adults admitted to the ICU or tertiary hospitals. We finally identified 5397 eligible patients. The number of patients receiving ≥ 3500 mL per day of IVF was 1679 (31.1%) and that of patients receiving < 3500 mL per day of IVF was 3718 (68.9%) (Fig. 1).

Table 1 presents the baseline patient characteristics. The total volume of IVF within 3 days from admission was 11,039 mL and 4054 mL in the IVF ≥ 3500 mL and < 3500 mL groups, respectively. Patients in the IVF ≥ 3500 mL group were younger than those in the IVF < 3500 mL group. The proportions of male, ICU admissions, receipt of mechanical ventilation, continuous renal replacement therapy, vasopressors, sodium bicarbonate, and diuretics in the IVF ≥ 3500 mL group were higher than those in the IVF < 3500 mL group. The proportions of tertiary hospital admission and use of lipid-lowering drugs in the IVF ≥ 3500 mL group were lower than those in the IVF < 3500 mL group. There were significant differences in body mass index, consciousness, and Charlson Comorbidity Index between the groups.

**Table 1:** Patient characteristics

Characteristics	Overall patients		Before overlap weighting					After overlap weighting				
			IVF ≥ 3500 mL/day		IVF < 3500 mL/day		ASD	IVF ≥ 3500 mL/day		IVF < 3500 mL/day		ASD
Age, years, mean (SD)	66.8	(17.5)	61.1	(17.7)	69.5	(16.7)	0.49	(17.2)		(18.1)		0.03
Age, years, Median (IQR)	71	(56, 80)	64	(49, 76)	73	(60,82)						
Male, n (%)	3512	(65.1)	1218	(72.6)	2294	(61.7)	0.23	(30.3)		(31.4)		0.02
Body mass index, kg/cm^2^, n (%)							0.21					0.04
< 18.5	710	(13.2)	168	(11.4)	542	(16.7)		(13.1)		(13.1)		
18.5–24.9	2729	(50.6)	843	(57.1)	1886	(58.2)		(57.5)		(58.9)		
25.0–29.9	982	(18.2)	334	(22.6)	648	(20.0)		(21.7)		(21.0)		
≥ 30.0	296	(5.5)	131	(8.9)	165	(5.1)		(7.8)		(7.0)		
Missing	675	(12.5)	201	(12.0)	474	(12.8)						
Japanese Coma Scale, n (%)							0.13					0.03
0 (alert)	2168	(40.2)	658	(39.2)	1510	(40.6)		(41.0)		(39.7)		
1-digit (dizziness)	2440	(45.3)	738	(44.0)	1702	(45.8)		(44.6)		(45.8)		
2-digit (somnolence)	519	(9.6)	165	(9.8)	354	(9.5)		(9.1)		(9.6)		
3-digit (coma)	265	(4.9)	116	(6.9)	149	(4.0)		(5.2)		(4.9)		
Charlson Comorbidity Index, n (%)							0.17					0.05
0	3925	(72.8)	1297	(77.3)	2628	(70.7)		(75.3)		(73.9)		
1	332	(6.2)	103	(6.1)	229	(6.2)		(6.6)		(6.6)		
2	923	(17.1)	227	(13.5)	696	(18.7)		(14.5)		(16.2)		
≥ 3	212	(3.9)	50	(3.0)	162	(4.4)		(3.5)		(3.3)		
Tertiary hospital admission, n (%)	4532	(84.1)	1269	(75.7)	3229	(86.9)	0.18	(83.0)		(84.8)		0.05
ICU admission, n (%)	946	(17.5)	399	(24.0)	547	(14.7)	0.23	(18.7)		(16.9)		0.05
Drug or device use (days 1–3), n (%)												
Mechanical ventilation	323	(6.0)	220	(13.1)	103	(2.8)	0.38	(6.6)		(5.9)		0.03
High-flow nasal cannula	11	(0.2)	8	(0.5)	3	(0.1)	0.08	(0.2)		(0.2)		0.01
Other oxygen device	2202	(40.8)	827	(49.3)	1375	(37.0)	0.25	(44.5)		(43.4)		0.02
Hemodialysis	549	(10.2)	425	(25.3)	124	(3.3)	0.66	(9.6)		(9.0)		0.02
Vasopressors	337	(6.3)	227	(13.5)	110	(3.0)	0.39	(6.4)		(6.1)		0.01
Sodium bicarbonate	313	(5.8)	154	(9.2)	159	(4.3)	0.20	(6.1)		(6.6)		0.02
Loop diuretics	894	(16.6)	408	(24.3)	486	(13.1)	0.29	(18.6)		(17.3)		0.03
Lipid-lowering drugs	269	(5.0)	45	(2.7)	224	(6.0)	0.16	(3.6)		(3.7)		0.00
Disease, n (%)												
Infection	737	(13.7)	229	(13.7)	508	(13.7)	0.00	(12.2)		(12.7)		0.02
Trauma	677	(12.6)	201	(12.0)	476	(12.8)	0.03	(11.8)		(12.0)		0.01
Epilepsy	206	(3.8)	67	(4.0)	139	(3.7)	0.01	(4.1)		(4.4)		0.01
Heatstroke	146	(2.7)	53	(3.2)	93	(2.5)	0.04	(3.0)		(2.9)		0.00
CKD	126	(2.3)	24	(1.4)	102	(2.7)	0.09	(2.1)		(2.1)		0.00
Hypothermia	133	(2.5)	48	(2.9)	85	(2.3)	0.04	(2.4)		(2.2)		0.01
Neuroleptic malignant syndrome	103	(1.9)	46	(2.7)	57	(1.5)	0.08	(2.0)		(2.2)		0.01
Hypothyroid	50	(0.9)	13	(0.8)	37	(1.0)	0.02	(0.8)		(0.8)		0.01
Burn	34	(0.6)	20	(1.2)	14	(0.4)	0.09	(0.7)		(0.7)		0.00
Drug intoxication	121	(2.2)	52	(3.1)	69	(1.9)	0.08	(2.8)		(2.8)		0.00
Ethanol intoxication	12	(0.2)	5	(0.3)	7	(0.2)	0.02	(0.2)		(0.2)		0.01
Diabetic ketoacidosis	9	(0.2)	5	(0.3)	4	(0.1)	0.04	(0.2)		(0.2)		0.00
Severe asthma	3	(0.1)	2	(0.1)	1	(0.0)	0.03	(0.1)		(0.1)		0.00
Maintenance fluid (days 1–3), mL, mean (SD)	1168	(7042)	808	(1453.9)	1331	(8423.5)	0.09	838	(1486)	995	(1470)	0.06
Maintenance fluid (days 1–3), mL, median (IQR)	0	(0, 2000)	0	(0, 1000)	500	(0, 2000)						

*ASD* absolute standardized difference, *CKD* chronic kidney disease, *ICU* intensive care unit, *IQR* interquartile range, *IVF* intravenous fluid, *SD* standard deviation

Table 2 presents the results of the crude outcomes. In the IVF ≥ 3500 mL and IVF < 3500 mL groups, the proportions of primary composite outcome were 5.1% (86/1677) and 2.7% (100/3715), respectively; hemodialysis dependence was 0.8% (14/1677) and 0.5% (18/3715), and in-hospital mortality were 4.3% (72/1677) and 2.2% (82/3715), respectively. The mean (SD) length of 28-day hospital-free days was 10.2 (9.3) and 12.5 (8.8) days in the IVF ≥ 3500 mL and IVF < 3500 mL groups, respectively. Among the patients receiving mechanical ventilation, the length of mechanical ventilation was 19.1 (8.4) and 20.3 (7.9) days, respectively. Among those who received oxygenation, the length of 28-day oxygenation free days were 20.0 (8.3) and 21.5 (7.4) days in the IVF ≥ 3500 mL group and the IVF < 3500 mL group, respectively.

**Table 2:** Crude outcomes and propensity score-adjusted outcomes between the ≥ 3500 mL IVF group and the < 3500 mL group

	Crude analysis		Propensity score analysis				
	≥ 3500 mL/day	< 3500 mL/day	≥ 3500 mL/day	< 3500 mL/day	RD	95% CI	P value
Primary composite outcome, n (%)	86 (5.1)	100 (2.7)	(3.4)	(3.9)	− 0.4	(− 1.8 to 0.9)	0.53
HD dependance at discharge, n (%)	14 (0.8)	18 (0.5)	(0.4)	(1.3)	− 0.9	(− 1.6 to − 0.2)	0.02
In-hospital mortality, n (%)	72 (4.3)	82 (2.2)	(2.5)	(0.5)	0.5	(− 0.7 to 1.6)	0.43
			Coefficients (95% CI)				
28-day hospital free days, days (SD)	10.2 (9.3)	12.5 (8.8)	11.2 (9.2)	12.5 (8.9)	− 1.3	(− 2.0 to − 0.7)	< 0.01

*IVF* intravenous fluid, *CI* confidence interval, *SD* standard deviation

Table 2 also presents the results of the propensity score-adjusted outcomes. There was no significant difference in the primary composite outcome (3.4% vs. 3.9%; risk difference, − 0.4; 95% confidence interval [CI] − 1.8 to 0.9). However, IVF ≥ 3500 mL was associated with reduced hemodialysis dependence at discharge (0.4% vs. 1.3%; risk difference, − 0.9; 95%CI − 1.6 to − 0.2). The IVF ≥ 3500 mL was not associated with reduced in-hospital mortality (3.0% vs. 2.5%; risk difference, 0.5; 95% CI, − 0.7 to 1.6). IVF ≥ 3500 mL was associated with a shorter length of 28-day hospital-free days (11.2 days vs. 12.5 days; difference, − 1.3 days; 95% CI − 2.0 to − 0.7).

There was no significant difference in the duration of 28-day ventilator-free days (19.5 days vs. 20.1 days; difference, − 0.6 days; 95%CI − 2.5 to 1.4) and duration of 28-day oxygenation-free days (21.1 days vs. 21.1 days; difference, − 0.1 days; 95%CI − 0.7 to 0.7) between the groups.

**Fig. 2:**
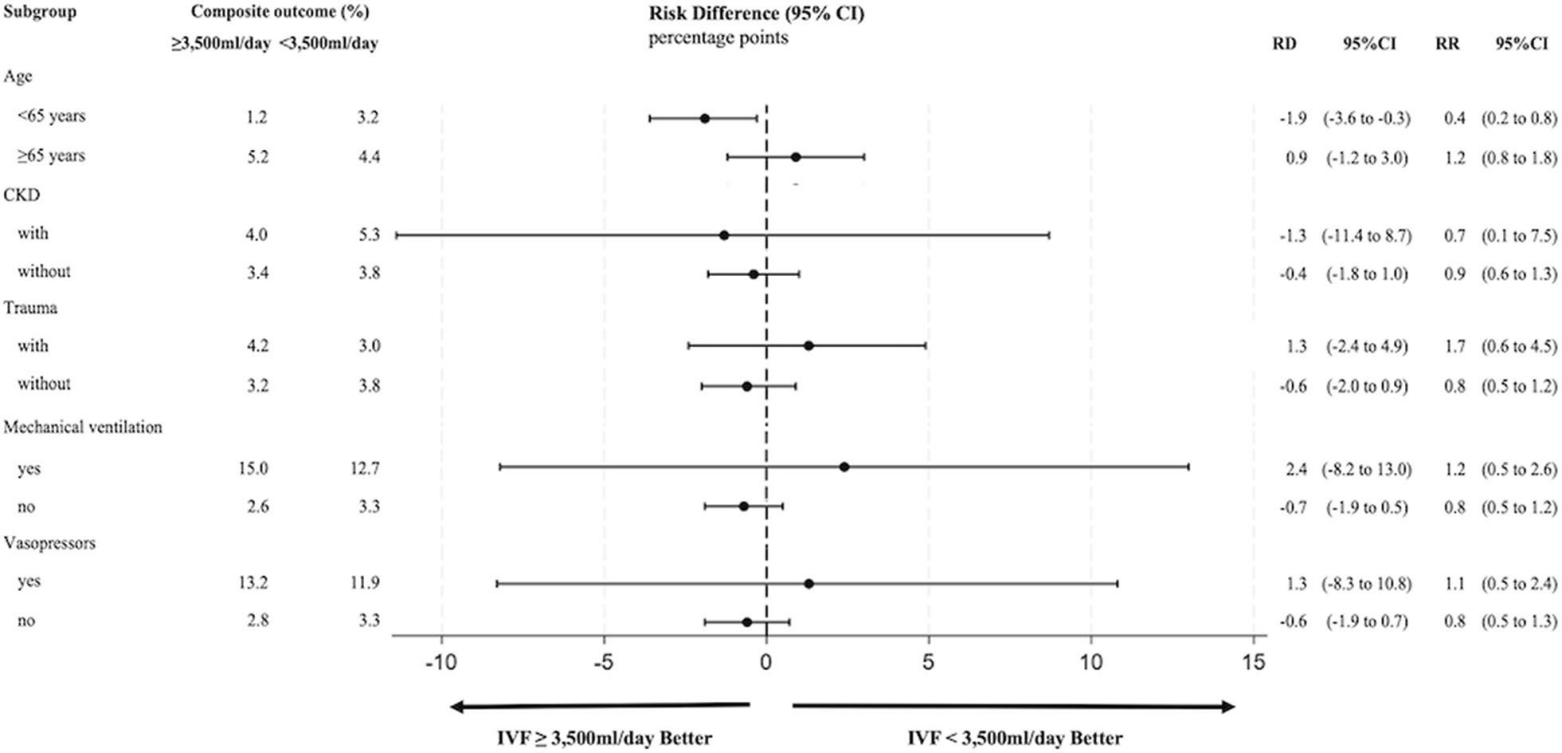
Forest plots of RDs of IVF ≥ 3500 mL/day for primary composite outcome in subgroups. *RD* risk difference, *RR* risk ratio, *CI* confidence interval, *CKD* chronic kidney disease. We conducted a propensity-score overlap weights for the overall population, and then we compared the risk difference within each subgroup

The results of the subgroup analyses are presented in Fig. 2 and Additional files 1 and 2. There was no interaction of the composite outcome, hemodialysis dependence, and in-hospital death within all subgroups. Additional file 3 presents the results of propensity matching weight analysis. The numbers (percentages) of patients receiving IVF < 3500 mL, 3500–5999 mL and ≥ 6000 mL were 3715 (68.9%), 1164 (21.6%) and 513 (9.5%), respectively. Compared with the IVF < 3500 mL group, the IVF 3500–5999 mL group showed a significantly higher proportion of the primary outcome (4.2% vs. 7.0%; RD, − 2.8; 95% CI − 5.7 to < − 0.01), and no significant difference with the IVF ≥ 6000 mL group was observed (5.5% vs. 7.0%; RD, − 1.6; 95% CI − 4.8 to 1.7). Compared with the IVF < 3500 mL group, both the IVF 3500–5999 mL and IVF ≥ 6000 mL groups had significantly lower proportions of hemodialysis dependence at discharge (0.3% vs. 3.4%; RD, − 3.0; 95% CI − 4.9 to − 1.2 and 1.1% vs. 3.4%; RD, − 2.3; 95% CI − 4.3 to − 0.2, respectively). No significant difference in in-hospital mortality was observed among the three groups. The results of other sensitivity analyses were shown in Additional files 4 and 5. The results were similar to those of the main analysis.

## Discussion

This study investigated the association between ≥ 3500 mL/day of IVF and the composite outcome of hemodialysis dependence at discharge or in-hospital death in patients with rhabdomyolysis. IVF ≥ 3500 mL was not associated with decreased composite outcome and in-hospital death. However, IVF ≥ 3500 mL was associated with reduced hemodialysis dependence at discharge and a longer hospital stay.

To the best of our knowledge, this is the first study to focus on the association between IVF volume and hemodialysis dependence upon discharge. The sample size was large, and we adjusted for many confounding factors. Initial IVF resuscitation was associated with renal protection, which was consistent with previous observational studies [5, 6]. The number needed to treat to prevent one case of hemodialysis dependence at discharge was 111.1 (95% CI, 66.7 to 500.0). AKI due to rhabdomyolysis accounted for 10% of all AKI cases [1]. Generally, the quality of life in patients with hemodialysis is relatively low and the costs of hemodialysis are high. Although the estimate of treatment effect in this study was small, initial IVF ≥ 3500 mL can be a feasible option for reducing hemodialysis dependence. Similar to previous studies, there was no significant difference in mortality due to fluid infusion [9].

In the present study, IVF ≥ 3500 mL was associated with shorter 28-day hospital-free days. In the IVF ≥ 3500 mL group, more IVF was administered and diuretics were used more frequently. Hospital discharge may have been delayed until total fluid balance normalized and tissue edema improved, resulting in a longer hospital stay.

In the sensitivity analyses, IVF 3500–5999 mL was associated with a significant reduction in primary outcome compared with IVF < 3500 mL. This results in the IVF 3500–5999 mL group may have demonstrated the proper balance between the renal protection of IVF and organ injury caused by excessive IVF. However, it was also possible that the severity of diseases was not adequately adjusted and critically ill patients localized in the IVF ≥ 6000 mL group, resulting in high mortality. The reduction in hemodialysis dependence at discharge was consistent with sensitivity analyses results in the IVF ≥ 3500 mL group.

This study has some limitations. First, data on CK levels, urine output, and the complication of AKI were not obtained. CK levels, urine output and the complication of AKI were confounding factors related to the diagnosis and treatment of rhabdomyolysis, which may have resulted in a lack of adjustment for disease severity and patient characteristics. We adjusted for the use of diuretics, vasopressors, mechanical ventilation, CKD on admission, and causes of rhabdomyolysis. It remains controversial whether levels of CK at admission are associated with the development of AKI [9, 22–24]. Second, severity scores, such as APACHE II and SOFA, were not available in our study. Although we adjusted for the severity of illness as much as possible through the use of diuretics, vasopressors, mechanical ventilation, CKD on admission, and causes of rhabdomyolysis, there was a possibility of unmeasured confounders. Third, the mortality in the present study was lower than that in previous studies [10, 25]. This may be because the diagnostic criteria for rhabdomyolysis were not standardized, and misclassification of the disease may have occurred in the present study, resulting in selection bias. Fourth, we could not evaluate any long-term improvements in renal impairment. We followed the patients only during hospitalization and were not able to determine their status after discharge. Patients may have been weaned from hemodialysis. Fifth, there are limitations due to the use of administrative claims database. For example, the sensitivity of diagnosis was not high, and it was difficult to identify hemodialysis before admission. Patients with CKD who were not dependent on hemodialysis before hospitalization may have been excluded. Moreover, patients with trauma from traffic accidents were not included in the DPC database because payment for most traffic accidents was not covered by the public health insurance system in Japan. Sixth, although we adjusted for many confounding factors by using the propensity score overlap weighting method, the influence of unmeasured confounding factors could not be fully excluded.

Although the optimal fluid volume remains unclear, our study showed that IVF ≥ 3500 mL can reduce hemodialysis dependence in patients with rhabdomyolysis. IVF ≥ 3500 mL may offer the potential for renal protection without increasing respiratory and renal complications. Future studies should investigate the improvement of the quality of life in patients receiving initial IVF ≥ 3500 mL and cost-effectiveness of initial IVF ≥ 3500 mL.

## Conclusions

Our study suggests that initial IVF ≥ 3500 mL/day may decrease dialysis dependence but did not decrease in-hospital death for patients with rhabdomyolysis. The optimal volume remains unknown, and further studies are warranted.

## Supplementary Information

Supplementary Material 1. Figure S1. Forest plots of RDs of IVF ≥ 3500 mL/day for hemodialysis dependence at discharge in subgroups.

Supplementary Material 2. Figure S2. Forest plots of RDs of IVF ≥ 3500 mL/day for in-hospital death in subgroups.

Supplementary Material 3. Table S1. Outcomes of propensity matching weight analysis between the IVF < 3500 mL, 3500–5999 mL, and > 6000 mL groups.

Supplementary Material 4. Table S2. Propensity score-adjusted outcomes between the IVF ≥ 60 mL/kg/day and IVF < 60 mL/kg/day groups.

Supplementary Material 5. Table S3. Propensity score-adjusted outcomes between the IVF ≥ 3500 mL/day and IVF < 3500 mL/day groups, taking into account the use of normal saline.

## Data Availability

Data will not be shared due to the policy of the institutional review board.

## Abbreviations

AKI: Acute kidney injury
CK: Creatinine kinase
CKD: Chronic kidney disease
ICD-10: International Classification of Diseases, Tenth Revision
IVF: Intravenous fluid
