# Supplementary material for: Association between initial intravenous fluid volume and the composite outcome of hemodialysis dependence at discharge or in-hospital mortality in inpatients with rhabdomyolysis
Source: J Intensive Care. 2025 Apr 27;13:22. doi: 10.1186/s40560-025-00788-w (PMC12034192; doi:10.1186/s40560-025-00788-w)
Supplement: Supplementary file 1 — Supplementary Material 1. Figure S1. Forest plots of RDs of IVF ≥ 3500 mL/day for hemodialysis dependence at discharge in subgroups. [file 40560_2025_788_MOESM1_ESM.docx]

Figure S1. Forest plots of RDs of IVF ≥ 3,500 mL/day for hemodialysis dependence at discharge in subgroups


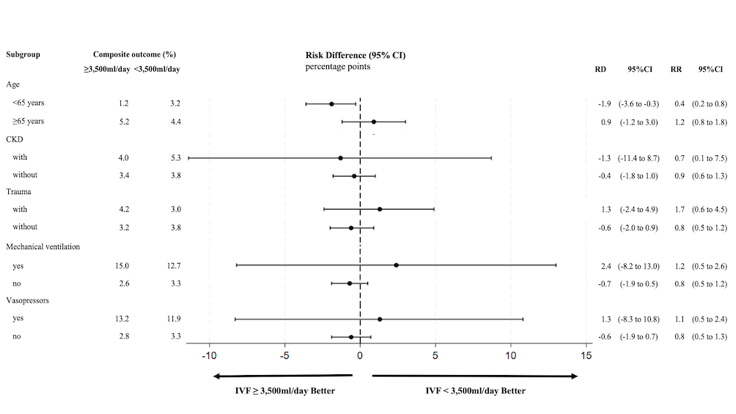


CI, confidence interval; CKD, chronic kidney disease; IVF, intravenous fluid; HD, hemodialysis; RD, risk difference
