# Supplementary material for: Association between initial intravenous fluid volume and the composite outcome of hemodialysis dependence at discharge or in-hospital mortality in inpatients with rhabdomyolysis
Source: J Intensive Care. 2025 Apr 27;13:22. doi: 10.1186/s40560-025-00788-w (PMC12034192; doi:10.1186/s40560-025-00788-w)
Supplement: Supplementary file 2 — Supplementary Material 2. Figure S2. Forest plots of RDs of IVF ≥ 3500 mL/day for in-hospital death in subgroups. [file 40560_2025_788_MOESM2_ESM.docx]

Figure S2. Forest plots of RDs of IVF ≥ 3,500 mL/day for in-hospital death in subgroups
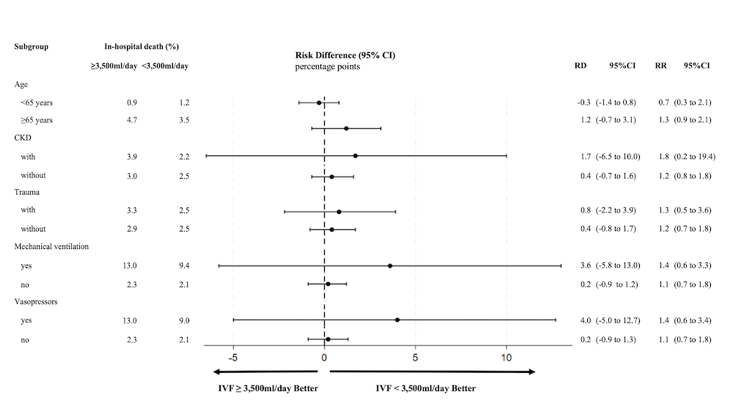


CI, confidence interval; CKD, chronic kidney disease; IVF, intravenous fluid; RD, risk difference
