# Supplementary material for: Association between initial intravenous fluid volume and the composite outcome of hemodialysis dependence at discharge or in-hospital mortality in inpatients with rhabdomyolysis
Source: J Intensive Care. 2025 Apr 27;13:22. doi: 10.1186/s40560-025-00788-w (PMC12034192; doi:10.1186/s40560-025-00788-w)
Supplement: Supplementary file 3 — Supplementary Material 3. Table S1. Outcomes of propensity matching weight analysis between the IVF < 3500 mL, 3500–5999 mL, and > 6000 mL groups. [file 40560_2025_788_MOESM3_ESM.docx]

Table S1. Outcomes of propensity matching weight analysis between the IVF < 3,500 mL, 3,500–5,999 mL, and > 6,000 mL groups

| Groups | Outcomes | Multivariable regression analysis | | |
| --- | --- | --- | --- | --- |
|  |  | Risk Difference (%) | 95% CI | P value |
| IVF | Primary composite outcome (%) |  |  |  |
| < 3,500 mL | 7.0 | (Reference) |  |  |
| 3,500–5,999 mL | 4.2 | -2.8 | (-5.7 to < -0.01) | 0.049 |
| ≥ 6,000 mL | 5.5 | -1.6 | (-4.8 to 1.7) | 0.35 |
| IVF | HD dependence at discharge (%) |  |  |  |
| < 3,500 mL | 3.4 | (Reference) |  |  |
| 3,500–5,999 mL | 0.3 | -3.0 | (-4.9 to -1.2) | < 0.01 |
| ≥ 6,000 mL | 1.1 | -2.3 | (-4.3 to -0.2) | 0.03 |
| IVF | In-hospital mortality (%) |  |  |  |
| < 3,500 mL | 3.6 | (Reference) |  |  |
| 3,500–5,999 mL | 3.8 | 0.2 | (-2.1 to 2.5) | 0.87 |
| ≥ 6,000 mL | 4.3 | 0.7 | (-2.0 to 3.3) | 0.60 |

IVF, intravenous fluid; RD, risk difference; CI, confidence interval; HD, hemodialysis
