# Supplementary material for: Association between initial intravenous fluid volume and the composite outcome of hemodialysis dependence at discharge or in-hospital mortality in inpatients with rhabdomyolysis
Source: J Intensive Care. 2025 Apr 27;13:22. doi: 10.1186/s40560-025-00788-w (PMC12034192; doi:10.1186/s40560-025-00788-w)
Supplement: Supplementary file 4 — Supplementary Material 4. Table S2. Propensity score-adjusted outcomes between the IVF ≥ 60 mL/kg/day and IVF < 60 mL/kg/day groups. [file 40560_2025_788_MOESM4_ESM.docx]

Table S2. Propensity score-adjusted outcomes between the IVF ≥ 60 mL/kg/day and IVF < 60 mL/kg/day groups

|  | ≥ 60 mL/kg/day | < 60 mL/kg/day | RD | 95% CI | P value |
| --- | --- | --- | --- | --- | --- |
| Primary Composite outcome (%) | 3.2 | 3.4 | -0.2 | ( -1.6 to 1.2) | 0.80 |
| HD dependance at discharge (%) | 0.4 | 1.2 | -0.8 | (-1.5 to -0.02) | 0.045 |
| In-hospital mortality (%) | 2.8 | 2.2 | 0.6 | (-0.6 to 1.8) | 0.33 |

CI, confidence interval; HD, hemodialysis; IVF, intravenous fluid; RD, risk difference
