# Supplementary material for: Association between initial intravenous fluid volume and the composite outcome of hemodialysis dependence at discharge or in-hospital mortality in inpatients with rhabdomyolysis
Source: J Intensive Care. 2025 Apr 27;13:22. doi: 10.1186/s40560-025-00788-w (PMC12034192; doi:10.1186/s40560-025-00788-w)
Supplement: Supplementary file 5 — Supplementary Material 5. Table S3. Propensity score-adjusted outcomes between the IVF ≥ 3500 mL/day and IVF < 3500 mL/day groups, taking into account the use of normal saline. [file 40560_2025_788_MOESM5_ESM.docx]

Table S3. Propensity score-adjusted outcomes between the IVF ≥ 3,500 mL/day and IVF < 3,500 mL/day groups, taking into account the use of normal saline

|  | ≥ 3,500 mL/day | < 3500 mL/day | RD | 95% CI | P value |
| --- | --- | --- | --- | --- | --- |
| Primary composite outcome (%) | 3.2 | 3.8 | -0.5 | ( -1.8 to 0.8) | 0.45 |
| HD dependance at discharge (%) | 0.4 | 1.3 | -0.9 | (-1.6 to -0.2) | 0.02 |
| In-hospital mortality (%) | 2.8 | 2.4 | 0.4 | (-0.7 to 1.5) | 0.48 |

CI, confidence interval; HD, hemodialysis; IVF, intravenous fluid; RD, risk difference
